# Reference values for T and B lymphocyte subpopulations in Turkish children and adults

**DOI:** 10.3906/sag-2010-176

**Published:** 2021-08-30

**Authors:** Özge BESCİ, Dilek BAŞER, İsmail ÖĞÜLÜR, Ayşe Cansu BERBEROĞLU, Ayça KIYKIM, Tolga BESCİ, Asım LEBLEBİCİ, Hülya ELLİDOKUZ, Perran BORAN, Eren ÖZEK, Goncagül HAKLAR, Ahmet ÖZEN, Safa BARIŞ, Elif AYDINER

**Affiliations:** 1 Department of Pediatric Allergy-Immunology, Faculty of Medicine, Marmara University, İstanbul Turkey; 2 Department of Pediatric Endocrinology, Faculty of Medicine, Dokuz Eylül University, İzmir Turkey; 3 Department of Pediatrics, Marmara University, Faculty of Medicine, İstanbul Turkey; 4 Department of Pediatric Intensive Care Unit, Faculty of Medicine, Dokuz Eylül University, İzmir Turkey; 5 Department of Translational Oncology, Faculty of Health Sciences, Dokuz Eylül University, İzmir Turkey; 6 Department of Biostatistics and Medical Informatics, Faculty of Medicine, Dokuz Eylül University, İzmir Turkey; 7 Department of Social Pediatrics, Faculty of Medicine University, Marmara University, İstanbul Turkey; 8 Department of Neonatology, Faculty of Medicine, Marmara University, İstanbul Turkey; 9 Department of Social Biochemistry, Faculty of Medicine, Marmara University, İstanbul Turkey

**Keywords:** Immunophenotyping, lymphocyte subsets, reference values, lymphocyte percentage, absolute count

## Abstract

**Background/aim:**

Established reference values are critical for the interpretation of immunologic assessments. In particular, the proportion and absolute counts of T- and B- cell subpopulations are subject to change with age and ethnicity. We aimed to establish age-specific reference values for lymphocyte subsets using updated immunophenotyping panels.

**Materials and methods:**

We studied a total of 297 healthy Turkish subjects aged 0 to 50 years, stratified into major age brackets in a cluster factor of 10 per age-group. The predetermined age intervals contained randomly allocated participants enrolled over a period of 6 months, who were homogenously distributed by sex. We analyzed a complete blood count test and simultaneously with detailed immunophenotyping enumerated the percent and absolute cell counts of lymphocyte subsets.

**Results:**

The percentage and absolute counts of lymphocyte subsets show a marked surge across the age-span. T helper, T cytotoxic, and the natural killer cell numbers were increasing from birth until 6 months, followed by a gradual decrease thereafter. B cell numbers were rising until 2 years, followed by a gradual decrease for the upcoming years, accompanied by a steady expansion of unclass-switched- and class-switched- B cells.

**Conclusion:**

We provide updated extensive reference intervals for lymphocyte subpopulations in Turkish people.

## 1. Introduction

The determination of cell-surface markers that define functional subsets of lymphocytes has immensely contributed to the understanding of the human immune system. The utilization of flow cytometry in the clinical lab and applications of immune-based staining to phenotype the major subsets have improved the diagnostic precision of the immunologists with regards to immunological and hematological disorders [1–11]. Lymphocyte subpopulation analysis studies are broadly used for diagnosis and classification of over 430 different inborn errors of immunity as well as monitoring the treatment outcome [12]. The distribution of lymphocyte subpopulations in a variety of immune-mediated diseases is a recent sparkling area of research, but the variability of normal ranges requires well-established reference values. 

Lymphocyte subset counts and percentages are significantly influenced by different environmental factors, antigen stimulation, ethnicity, vaccination schedule, and the characteristics of the flow cytometry laboratory [1–3,5–11,13]. Further, immune markers are in evolution and require updates to reflect the current understanding of lymphocyte biology. Therefore, reference determination is a dynamic process, in which population-specific references should be periodically updated to account for this evolution. Former studies have successfully determined the composition of T- and B-cell pools, yet few of them conducted a systematic approach to determine the distribution of each subset from birth to the entire childhood span [1–3,5–11]. The most recent reference determination study for the Turkish population was published in 2004, however, new immunologic subsets have been defined and implemented into medical practice since then, warranting updated investigation into the subject [3].

The current study establishes age-dependent reference values of lymphocyte subsets for both children and adults, with an updated panel that expands the repertoire of the examined subsets over the former work. We think that our results would be useful for both immunology and allergy specialists as well as physicians during the evaluation of primary and secondary forms of immune deficiencies.

## 2. Materials and methods

### 2.1. Subjects and samples

This is a cross-sectional study involving healthy children and adults. Peripheral blood samples were provided from children aged 0 through 18 years who were seen in our general pediatric clinics, well-child outpatient clinics (<5 years of age). The cord blood was obtained from newborns who were delivered in the Marmara University Research and Training Hospital’s Obstetrics Department. The adult volunteers who provided blood samples were the family members of pediatric participants. Ethical approval was obtained from Marmara University Medical Faculty Clinical Researches Ethics Committee with the number of 09.2015.239/70737436-0.50.06.04. Eligibility criteria included: any children or adults in good health; full-term newborn (39–41 gestational); children within the normal range of growth indices (in between 3–97 percentile). The eligibility was assessed through history taking and physical examination to exclude any current or chronic diseases or conditions that may interfere with the results of the study. The volunteers filled out a questionnaire concerning their health and they were considered as clinically healthy if they did not have fever, respiratory or gastrointestinal disease during the last 2 weeks, no medication use other than vitamins within the last 2 weeks. Individuals with chronic diseases, active infections, or individuals who are taking any kind of medications, or those with immune-deficient relatives were excluded from the study. Their immunizations were questioned, and those who did not receive a vaccine in the last month prior to blood withdrawal were included. For the newborns, exclusion criteria also included chorioamnionitis, premature rupture of membrane, chronic illness of the mother, in utero malnutrition, Rh incompatibility, signs of infection, suspicion of hematological and neurological diseases, neonatal malformation, and dysmorphic features. Blood draw was performed following informed consent, either from the subjects themselves (adult subjects) or from the parents of the pediatric participants.

The minimum number of 30 attendees in each age stratum was determined based on previous studies and the law of great numbers [1–3,5–11] We aimed to study equal numbers from each sex for every age bracket. Out of 307 individuals enrolled in the study, 49.5% (147) were females and 50.5% (150) males; with the total number of subjects permissive for data analyses being 297. 

Samples were distributed into 10 age strata: cord blood (after clamping the umbilical cord at birth), 0 to 40 days (from declared as birth to 39 days old), 40 days to 6 months (from 40 days old to 180 days old), 6 to 9 months (from 181 to 270 days old), 9 to 12 months (from 271 to 365 days), 1 to 2 years (from 366 days to the second birthday), 2 to 5 years (from the day after the 2nd birthday to the 5th birthday), 5 to 10 years (from the day after the 5th birthday to the 10th birthday), 10 to 16 years (from the day after 10th birthday to the 16th birthday), after 16 years (from the day after 16th birthday to 50 years). The number of samples form participants (n) for each stratum was as follows: neonatal cord blood (n = 29), 0–40 days (n = 33), 40 days-6 months (n = 29), 6–9 months (n= 34), 9–12 months (n = 29), 1–2 years (n = 30), 2–5 years (n = 29), 5–10 years (n = 34), 10–16 years (n = 30), >16 years (n = 30). Out of 620 data subsets, 86% reached the number equal to or higher than 28 and 74% to the number which is higher than 29.

### 2.2. Immunophenotyping

Peripheral blood from the antecubital region or dorsum of the hand or umbilical cord was taken in an amount of 0.5 mL (for complete blood count) and 2 mL (for flow cytometry) into dipotassium ethylenediaminetetraacetic acid tubes. For further contribution to the homogeneity of the sampling data, blinded specimens were studied with routine procedures for immunophenotyping and complete blood count analyses. Leucocyte differential including, absolute lymphocyte count was quantified by a complete blood count autoanalyzer (Unicell DxH 800, Beckman Coulter ABD). Relative percentages of T-, B-, and NK- cells, and the T- and B- cell subsets were measured using flow cytometry, which was then used to calculate the absolute counts of respective immune subsets. The staining of samples with an appropriate fluorochrome, lysing (BD FACS Lysing Solution, USA) and washing (BD, USA) performed simultaneously with CBC analysis. Previously optimized and standardized CD markers utilized: CD4-FITC/CD8-PE/CD3-PerCP, CD3-PerCP, CD4-PE, CD8-APC, TCR alfa/beta-FITC, CD45RA-FITC/ CD45RO-PE/ CD3-PerCP/ CD4-APC, CD45RA-FITC/ CD45RO-PE/ CD3-PerCP/CD8-APC, CD3-PerCP/ TCR alpha-beta-FITC/TCR gamma-delta-PE, CD14-PerCP, CD31-PE, CD27-APC, CD197(CCR7)-FITC, CD45RA-FITC, CD45/RA-PE, and CD8-PerCP for the T cells and their subsets; CD19-PerCP, CD3-FITC/CD19-PE, CD20-APC, CD21-PE, CD27-APC, CD38-APC, and IgD-PE for the B cells and their subsets, CD3-FITC/ CD16+56-PE for the NK cells. All antibodies were purchased from Becton Dickinson (San Jose, USA). An additional table defining the lymphocyte subsets with CD markers is presented in Table 1 [14]. Flow analysis was performed by BD Calibure and quadrant statistics were made by BD FACS CellQuest Pro software. Following the gating process performed by CD45 positivity and CD14 negativity for all leucocytes and the explained markers above for particular subsets, histograms indicated the percentage of different cell types after the correction. Absolute counts of different lymphocyte subtypes were calculated by (leucocyte count × % lymphocyte × % antibody positivity)/10,000 = Absolute number/μL formula [15].

**Table 1:** Lymphocyte subsets with CD markers.

Subsets	Expressed CD markers
Total T cell	3/19–
Total B cell	3–/19
Natural killer cell (NK)	16/56
Helper T cell (TH)	3/4
Cytotoxic T cell (TC)	3/8
Naive T cell	45RA
Memory T cell	45RO
Naive B cell	27–/IgD
Unclass-switched B cell	27/IgD
Class-switched B cell	27/IgD–
Plasmablast	38/IgM–
Autoreactive B cell	21low38low
Naive cytotoxic & helper T cells	CCR7/8/45RA & 4/45RA/27
Central memory cytotoxic & helper T cells	CCR7/8/45RA– & 4/45RA–/27
Effector memory cytotoxic & helper T cells	CCR7-/8/45RA– & 4/45RA–/27–
Terminal effector memory cytotoxic & helper T cells	8/45RA/CCR7– & 4/45RA/27–
TCR alpha-beta cells	TCR alpha-beta/3
TCR gamma-delta cells	TCR gamma-delta/3
Double negative T cells (DNT)	3/4–/8–
MHC class II	MHC class II
Recent thymic emigrant cells (RTE)	4/45RA/31

### 2.3. Statistical analysis

SPSS (Statistics Program for Social Sciences) program (v: 24.0) and Microsoft Excel were used for storage and analysis of data. Our assumption for defining reference values is that almost all of our data should be embraced concerning the sample size, mean, standard deviation, and confidence interval; in this sense, tolerance interval gives limits with specified confidence that a specified proportion of the population lies within these limits, was chosen as a method of analysis [7,16]. The upper and lower limits of the two-sided tolerance interval were set so that it enclosed at least 90% of the population, with a 95% of confidence interval. Shapiro–Wilks normality test and descriptive statistical histograms were used to define the homogeneity of values. On the other hand, most of our data were positively skewed like the previous studies so the logarithmic transformation was used to reduce skewness and normalize distribution for better statistical purposes [7]. Yet, even after logarithmic transformation some values were not normally distributed so nonparametric test results were calculated to define limit values. Both original and retransformed logarithmic data along with parametric and nonparametric limits indicated by asterisks are given in Tables 2–6. 

**Table 2:** Percentages and counts for peripheral blood lymphocyte subsets are presented for age intervals. Among the number of participants (n), median (med), lower and upper tolerance interval limits (Tilo-Tiup) of 90% range with 95% confidence level are calculated.

	Cord blood	0-39d	40d-≤6mo	6-≤9 mo	9-≤12 mo	1-≤2 yr	2-≤5 yr	5-≤10 yr	10-≤16yr	>16yr
n	29	31	31	34	28	31	29	34	30	30
ALC/mm3 (med)	4300	4850	5500	6900	5500	6200	4200	3100	2500	2100
Tilo-Tiup	2979–5490	2279–7592	2416–9694	3325-9563	2965-10471*	1829-10242	1703-6738	1803-5636	1403-4742*	1400-7100**
n	29	31	31	34	28	31	29	34	30	30
T cells (CD3+) % (med)	68.8	68.8	65.8	67.3	68	65.6	70	69.7	71.2	74.9
Tilo-Tiup	55.3–83.7	56.8–83	50.4–79.6	49.7-83	53.6–80.7	51-81.8	57.6-81.2	55-86.2	57.8-86.2	64.4-85
n	29	31	31	34	28	31	29	34	30	30
T cells (CD3+) # (med)	2873	3380	3899	4469	3672	3998	3074	2232	1837	1631
Tilo-Tiup	1850–4034	1765–5103	1492–6385	1981-6564	1945–7129*	1338-6611	1200-4706	971-3685	1032-3303*	998-5625**
n	29	31	31	34	28	31	29	34	30	30
Helper T (CD3+ CD4+) % (med) cells %	49.6	50.7	44.8	44.3	42.8	40.7	38.3	35.8	36.8	44.9
Tilo-Tiup	36.8–63.7	39.2–63.6	31.6–57.9	28.6-59.7	30–55.8	27.6-55.6	23.6-52.5	23.4-48.7	27.3-46.7	31.7-57.6
n	29	31	31	34	28	31	29	34	30	30
Helper T (CD3+ CD4+) cells # (med)	1989	2424	2750	2879	2166	2361	1498	1132	926	974
Tilo-Tiup	1330–3289*	1248–3779	909–4523	1190-4481	1161-4819*	820-4138	458-2755	445-1918	505-1778*	673-3110**
n	29	31	31	34	28	31	29	34	30	30
Cytotoxic T (CD3+CD8+) cells % (med)	18.4	17	20.5	19.4	21.6	21.4	23.8	27.7	27.9	25.3
Tilo-Tiup	8.5–28.5	9.8–26.7	10.7-28.2	9-31	11-33	12.7-30.9	12.1-35.7	16.8-46.5*	16.5-39.4	13.9-39.1
N	29	31	31	34	28	31	29	34	30	30
Cytotoxic T (CD3+CD8+) cells # (med)	783	880	1116	1125	1272	1201	986	884	680	563
Tilo-Tiup	391–1157	282–1525	254–2123	576-2582*	310-2250	540-2812*	165-1878	379-2084*	381-1312*	238-1570*
n	29	31	31	34	28	31	29	34	30	30
B cells (CD19+) % (med)	16.4	16	22.2	22.7	22.6	22.2	17.5	13	11	9.4
Tilo-Tiup	5.9–26.5	2.6–30.1	10.2–36	5.4-39.6	9.1-35.9	11-34.2	8.4-28.5	6.5-20.3	5.1-21.9*	3.4-15.9
n	29	31	31	34	28	31	29	34	30	30
B cells (CD19+) # (med)	749	650	1354	1383	1294	1352	736	396	260	222
Tilo-Tiup	180-1206	212-2244*	237-2564	117-2845	467-3112*	516-3083*	205-1341	122-755	94-793*	87-541*
n	29	31	31	34	28	31	29	34	30	30
NK (CD16+56+) % (med)	5.9	8.7	6.5	6.5	7	6.8	7.3	9.8	13.6	11.5
Tilo-Tiup	2.9-13*	3-20*	1.8-27.4*	0.3-13.5	2.5-17.9*	2-26.3*	3.5-22.2*	4-29*	1.8-26.6	5.1-24.7*
n	29	31	31	34	28	31	29	34	30	30
NK (CD16+56+) #	240	396	384	386	385	413	336	353	373	248
Tilo-Tiup	130-512*	126-1130*	101-1633*	156-968*	130-1073*	101-1741*	88-1393*	105-1107*	94-1175*	91-766*

Abbreviations: d: days, mo: months, yr: years ALC: absolute lymphocyte count, NK: natural killer cells *Two-sided parametric tolerance interval of log-transformed data **Two-sided nonparametric tolerance interval of log-transformed data.

**Table 3:** Percentages for naive and memory populations of helper and cytotoxic T lymphocytes are presented for age intervals. Among the number of participants (n), median (med), lower and upper tolerance interval limits (Tilo-Tiup) of 90% range with 95% confidence level are calculated.

	Cord blood	0-39d	40d-≤6mo	6-≤9 mo	9-≤12 mo	1-≤2 yr	2-≤5 yr	5-≤10 yr	10-≤16yr	>16yr
n	29	31	31	34	28	31	29	34	30	30
CD4+ memory T cells (CD4+CD45RO+) % (med)	18.2	19	16.5	18.7	19.6	23	26	39.4	47.1	54.8
Tilo-Tiup	7.1–52.7*	9.5–41*	4.8–31.7	8.9–37.7*	9.6–30.8	11.5–34.4	12.8–42.5	24.6–56	28.2–67.6	28.2–86.6
n	29	31	31	34	28	31	29	34	30	30
CD4+ naive T cells (CD4+CD45RA+) % (med)	68	74.9	71.9	74.9	75	74.8	69.7	59.9	52.1	42.5
Tilo-Tiup	18.2–85.3**	44.8–83.5**	51.9–93.7	58.4–95.2	62.9–87.8	56.1–91	54.9–83.1	41.7–77.8	13–68.2**	8.2–73.3
n	29	31	31	34	28	31	29	34	30	30
CD8+ memory T cells ( CD8+CD45RO+) % (med)	16.3	13	17.9	21.5	18.6	21.8	20.2	30	37.1	40.2
Tilo-Tiup	5.9–45.2*	3.3–25.3	5.2–58.3*	4.2–38.8	5.4–60.7*	10.9–49.8*	9.4–52.1*	5.1–59.1	12–66	11.5–72.5
n	29	31	31	34	28	31	29	34	30	30
CD8+ naive T cells (CD8+CD45RA+) % (med)	75.4	78.9	74.5	71.3	70.9	73.3	79.1	62.8	57.9	51.4
Tilo-Tiup	57.5–92.7	66.4–92.3	39.7–97.5	49.6–99	41.6–99.1	46.6–92.2	34.3–90.3**	36–87.8	28–86.2	19.3–86.2

**Table 4:** Percentages and counts for peripheral B lymphocytes for age intervals are presented. Among the number of participants (n), median (med), lower, and upper tolerance interval limits (Tilo-Tiup) of 90% range with 95% confidence level are calculated.

	Cord blood	0-39d	40 d-≤6 mo	6-≤ 9mo	9-≤12mo	1-≤2yr	2-≤5yr	5-≤10yr	10-≤16yr	>16yr
n	29	31	31	34	27	30	29	34	30	30
Naive B cells (CD19+CD27- IgD+) % (med) )))gD+)(CD19+CD27-IgD+) %	89.3	86.9	90.7	88.1	85.6	82.2	75	66.3	67.6	56.8
Tilo-Tiup	82.1–96.3	77.–-95.5	78–96.4**	70–94.7**	71.9–93.1**	68.6–96.5	58–86.5**	45–84.5	43.6–87.8	33.7–79.2
n	29	31	31	34	27	30	29	34	30	30
Naive B cells (CD19+CD27-IgD+) # (med)	691	560	1146	1241	969	1041	553	267	176	119
Tilo-Tiup	138–1107	178–2004*	169–2354	365–3516*	355–2938*	417–2692*	192–1429*	40–536	51–615*	48–304*
n	29	31	31	34	27	29	29	34	30	30
UCS B cells (CD19+CD27+IgD+) % (med)	7.4	5.6	4.9	6.5	7.3	8.4	12	12.9	12.1	18.1
Tilo-Tiup	1–13.7	0.5–14**	0.9–9	3–12.8*	3.2–14.4*	1.6–16.7	4.8–20.5	3.6–24.2	2.4–22.3	5.3–31.6
n	29	31	31	34	27	29	29	34	30	30
UCS B cells (CD19+CD27+IgD+) # (med)	44	36	62	81	76	128	83	59	32	36
Tilo-Tiup	0.4–96	7–193*	17–212*	30–216*	32–206*	32–362*	38–202*	6–114	9–107*	11–127*
n	29	31	31	34	27	29	29	34	30	30
CS B cells (CD19+CD27+IgD-) % (med)	0.8	2.6	2.5	3.4	5.3	5.8	9.6	14.4	15.5	21.9
Tilo-Tiup	0.2–2.6*	0.2–7.3**	0.8–8.7*	0.4–12.9**	1.4–14.5*	2–16.6*	2.9–31.9*	6.7–31.1*	2.7–29	5.9–34.5
n	29	31	31	34	27	29	29	34	30	30
CS B cells (CD19+CD27+IgD-) # (med)	5	17	37	43	52	77	72	59	39	44
Tilo-Tiup	2–16*	4–88*	8–146*	12–138*	20–151*	23–247*	27–178*	24–148*	14–117*	10–171*
n	27	31	31	34	26	31	29	34	30	30
AR B cells (21low 38low) %(med)	3	2	2	2	3	3	4	5	5	4
Tilo-Tiup	0.3–6.3**	0.4–9.4*	0.5–5*	0.8–6.3*	0.5–5	0.9–9*	0.9–13.2*	1.8–14.7*	1.4–14.6*	1.2–14.2*
n	27	31	31	34	26	31	29	34	30	30
AR B cells (21low 38low) #	16	15	24	33	33	34	24	21	14	9
Tilo-Tiup	5–58*	3–61*	6–75*	8–110*	10–96*	11–118*	6–96*	8–55*	3–49*	2–32*

Abbreviations: d: days, mo: months, yr: years, UCS: unclass-switched CS: class-switched AR: Autoreactive *Two-sided parametric tolerance interval of log-transformed data **Two-sided nonparametric tolerance interval of log-transformed data.

**Table 5:** Percentages for subpopulations of CD4+ and CD8+ T cells are presented for age intervals. Among the number of participants (n), median (med), lower and upper tolerance interval limits (Tilo-Tiup) of 90% range with 95% confidence level are calculated.

	Cord blood	0-39d	40 d-≤6 mo	6-≤ 9mo	9-≤12mo	1-≤2yr	2-≤5yr	5-≤10yr	10-≤16yr	>16yr
n	29	31	31	33	27	31	29	34	30	28
Central memory CD4+ T cells(CD4+CD45RA-CD27+) % (med)	8.4	13.2	12	12.6	16.7	16.6	25.1	30.2	36.4	44.3
Tilo-Tiup	4.7–21.7**	3.4–24	2.4–23	0.2–26.5	3.3–30.2	10–36.5**	9.2–40.2	14–49	24.2–51.4	21.7–67
n	29	31	31	33	27	31	29	34	30	28
Naive CD4+ T cells(CD4+CD45RA+CD27+) % (med)	87.5	83.7	82.9	78.8	74	74.9	66.7	56.9	45.1	40
Tilo-Tiup	71.6–94.4**	70.6–94.4	64.4–97.3*	61.2–96.2	57.4–93	51.493	47.3–86.6	38.2–75	28.9–66.6	13.9–66.4
n	29	30	31	32	27	31	29	34	30	28
Effector memory CD4+ T cells(CD4+CD45RA-CD27-) % (med)	0.5	0.7	1.4	1.1	1.9	2	2.7	6.1	8.9	8.3
Tilo-Tiup	0.03–9.2*	0.09–7.1*	0.2–9.4*	0.2–7.2*	0.5–8*	0.3–10.9*	0.8–10.8*	2–16.2*	3.5–23.6*	2.9–24.6*
n	29	29	30	33	26	31	29	34	30	28
Terminal effector memory CD4+ T cells(CD4+CD45RA+CD27-) % (med)	0.6	1.1	1.3	4.1	3.2	4.2	2.5	3.4	3.2	4.4
Tilo-Tiup	0.06–18.3**	0.3–10.4**	0.06–50.3*	0.3–43.6*	0.2–44.5*	0.3–56.2*	0.1–41.2*	0.2–43.8*	0.3–26.2*	0.3–44.6*
n	29	31	28	31	28	28	26	33	27	26
Central memory CD8+ T cells(CCR7+CD8+CD45RA-CD27+) % (med)	6.4	6.8	2.5	3	2.8	2.6	3	3.9	5.2	6.5
Tilo-Tiup	1.9–25.5*	1.9–22*	0.3–6.4**	0.1–6.3**	0.3–5.2**	0.8–9.7*	0.9–9.4*	0.3–8.3	1.8–14.2*	1.4–27.9*
n	29	31	29	31	28	28	26	33	27	26
Naive CD8+ T cells(CCR7+CD8+CD45RA+CD27+) % (med)	75.4	77.3	62.5	56.4	57.3	50	52.6	38	35.2	34.4
Tilo-Tiup	57.8–93.1*	58.4–95.6	27.9–94.8*	29.4–87.6	19.4–92.7	21.5–78.9	19–82.7	17.5–62.2	11.4–61.4	4.1-67.5
n	29	31	28	30	27	28	26	33	27	26
Effector memory cells CD8+ T(CCR7-CD8+CD45RA-CD27+) % (med)	3	2.5	9.5	9.3	10.2	11.2	12.2	17.2	22.3	22.1
Tilo-Tiup	0.3–9.3**	0.8–9.7*	0.9–35.5**	0.1–25**	2.6–40*	3.2–38.7*	4–42.5*	2.1–33.3	6.4–37.6	0.7–39.9*
n	29	31	29	31	28	28	26	33	27	26
Terminal effector memory CD8+ T cells(CD8+CD45RA+CD27-) % (med)	12.4	9.2	24.3	26.2	26.6	30.9	27.7	36.8	36	31.9
Tilo-Tiup	1.1–24.2*	2.7–39.1*	2–54.6**	9.4–70.3*	8.8–77.3*	8.3–58.5	5.5–55.5	14.6–61.7	6.2–65.6	3.6–66

**Table 6:** Percentages for other cell populations are presented for age intervals. Among the number of participants (n), median (med), lower and upper tolerance interval limits (Tilo-Tiup) of 90% range with 95% confidence level are calculated.

	Cord blood	0-39d	40d-≤6 mo	6-≤ 9 mo	9-≤ 12mo	1-≤ 2 yr	2-≤ 5 yr	5-≤10 yr	10-≤16yr	>16yr
n	29	31	31	34	28	31	29	34	30	30
TCR αβ/CD3+	97.6	96.9	95.1	95.4	94.4	92.9	90.7	88.6	89.1	93.4
Tilo-Tiup	94.7–98.6**	92.5–99*	90.3–99	88.9–97.5**	86.7–97**	87–98.5	80–96.7**	68–96**	78.4–98.5	87–99.3
n	29	31	31	34	28	31	29	34	30	30
TCR γδ/CD3+ % (med)	2	3	5	4	5	7	9	11	11	6
Tilo-Tiup	1.1–4.9*	1.2–7.2*	0.9–9	2–10*	2.3–13.5*	1.5–12.2	3.4–19.9**	4.7–29.6*	1.5–21	0.6–12.3
n	29	31	31	34	28	31	29	34	30	30
RTE (CD4+CD45RA+CD31+) % (med)	72	70	74	76	72	69.5	63	54	44	31.5
Tilo-Tiup	56.2–90.3	57.5–84.1	56.6–90.7	62.2–88.6	56.5–87.4	42.5–82.2**	47.9-77	38.6–65.8**	25.2–63.7	10–56.7
n	29	31	31	34	28	31	28	33	29	30
DNT% (med)	0.7	0.7	0.7	0.9	1.2	1.7	2	2.3	1.8	1.4
Tilo-Tiup	0.07–1.3	0.2–2.6*	0.2–2*	0.3–2.4*	0.5–2.7*	0.2–2.9	0.8–3.2	0.2–4.5	0.4–3.4	0.5–3.9*

Abbreviations: d: days, mo: months, yr: years, TCR: T-cell receptor, RTE: recent thymic emigrants, DNT: double-negative T cells, αβ:alpha-beta, γδ: gamma-delta *Two-sided parametric tolerance interval of log-transformed data **Two-sided nonparametric tolerance interval of log-transformed data.

Statistical analysis to measure the significance of the differences between each age stratum for all 35 different peripheral blood lymphocyte subgroups was also performed. Homogeneity of variances was compared by Levene’s test, which indicated variance heterogeneity (p < 0.05). A Welch one-way analysis of variance (ANOVA) was applied to all 35 different peripheral blood lymphocyte subgroups to verify the statistical difference among the 10 age groups. Further, to compute the multiple pairwise comparison between the individual means of each age group, for which the homogeneity of variance has not been met, Games–Howell post hoc test was applied.

## 3. Results

We determined the percentage of lymphocyte subsets and calculated absolute cell counts as presented in Tables 2–6. We calculated the median and the lower and upper limits for each immune subset for every age-stratum. 

One-way ANOVA analysis showed the age difference was statistically significant (p-values < 0.05) for all 35 different lymphocyte subgroups, except for the terminal effector memory CD4+ T cells. Further, the Games–Howell post hoc test results showed that most individual age groups were also statistically different in their mean values (data not presented).

The absolute cell counts of CD3+ T lymphocytes gradually increased from birth up until 6 to 9 months when a maximum is reached (age-specific median = 4469/mm^3^ which then declines to values that are below the newborn levels (Table 2). We found that the absolute cell counts of CD4+ and CD8+ T lymphocytes, as well as CD45RA+ naive T cells follow a similar pattern, with a gradual rise until 9 months followed by a linear reduction over the subsequent ages (Tables 2 and 3). When investigating the trends in relative percentages, the CD3+, CD4+, and CD8+ T cells all showed stable characteristics at ranges between 65.6%–74.9%, 35.8%–50.7%, and 17%–27.9%, respectively (Table 2). CD19+ B cells doubled at 1 to 2 years of age interval which decreased by half after 2 to 5 years of age and remained stable throughout the life span. The relative percentage rates of the medians of B cells were distributed between 9.4%–22.7% (Table 2). Increasing NK-cell counts until the first 6 months showed a continuous decline afterward and their relative percentages increased to adulthood, however, a significant change pattern was not noted (Table 2). 

The percentages of central memory and effector memory CD4+ T cells tended to increase during childhood in contrast to naive CD4+ and CD8+ T cells which decreased (Table 5). 

Absolute cell counts of naive B cells tended to increase in the first 6 months followed by a decrease thereafter; this pattern was just in contrast to that of unclass-switched B cells. The absolute number of class-switched B cells increased in the first 2 years, then started decreasing; however, the percentage values for these cells were on a continuous rise. Autoreactive B cells (CD21lowCD38low) remained relatively stable throughout the lifespan at a fixed ratio of 2%–5% order. Autoreactive B cells that were relatively high at birth decreased in number till 40 days, then increased up to 2 years of age and showed a decrease again after 2 years of age (Table 4).

CD3+ TCR alpha-beta cells increased through adulthood; by contrast, the CD3+ gamma-delta cells decreased in percentage. The percentage of double-negative T cells (DNT) remained in the range of 0.2%–5.5% with no age-specific pattern. The percentages of recent thymic emigrants (RTE) cells increased in the first 9 months but then decreased gradually.

## 4. Discussion

In this study, we established the normative reference ranges for peripheral blood lymphocyte subsets in a healthy Turkish population. The investigated immune subsets included an updated profile over the former studies that examined subjects in the same region. We think that these references will be useful for interpreting the immunologic subsets, that are often ordered for the evaluation of immune-mediated diseases. 

The methodology we employed in this study was similar to previous work, in which lymphocyte subsets were investigated with respect to varying ages [1–3, 5–11]. The trend in the absolute numbers of CD3+ T lymphocytes, cytotoxic T cells, helper T cells was similar to İkincioğulları et al. [3] and Schatorjé et al. [7] with a gradual rise during the first months of life, followed by a progressive decline in the subsequent years. This observation was in contrast to Comans–Bitter et al. [1] and Tosato et al. [10] studies. Major differences were observed among the subpopulations of these cells. We observed a general trend in the cells with regards to their differentiation into memory phenotype: while the percentage of the naive compartment of the helper and cytotoxic T cells showed a negative trend with increasing age, the memory cells were increasing in relative size as the subjects grew to adults. This is unsurprising as one could expect that immune maturation reflects exposure to microorganisms over time [10]. Likewise, naive CD4+ and CD8+ T cells decreased by half in the lymphocyte maturation cycle. In contrast to Schatorjé et al.’s findings [7], which showed stable values for the size of the central memory pool of CD4+ T cells, in our study, the percentages were threefold higher in adulthood compared to infancy period. The effector and terminal effector memory pools of CD4+ and CD8+ T cells showed a significant rise with advanced age. Possible lymphocyte development defects have not emerged only with deficiencies but also with unexpected growth of specific clones. Therefore, an exaggerated increase in memory T-cells would be an alarming finding for T-cell dysfunction [11]. Furthermore, all these subgroups are especially important for newborns too, as for which hypomorphic defects or the presence of maternal engraftments could easily mislead the diagnosis [11]. Therefore, the standard immunophenotyping studies must be carefully examined in the light of expected ranges.

DNT cells, which are related to autoimmunity and one of the diagnostic criteria for the autoimmune lymphoproliferative disorder, varied in the range of 0.2% to 5.5% among all age groups [17]. This finding contrasts with the former reports which describe greater variations [7,10]. RTE cell production correlates with thymic output and is an important marker for diseases such as severe combined immunodeficiencies, rheumatoid factor-negative polyarticular juvenile idiopathic arthritis, systemic lupus erythematosus, psoriasis, type 1 diabetes have defects in T-cell development in the thymus [7]. Similar to the findings of Schatorjé et al. [7] the relative size of this group was increasing with age followed by a gradual regression. 

Within the B-cell pool, all of the examined subsets including class-switched B cells, unclass-switched B cells, and CD21lowCD38low B demonstrate a rise in number during the infancy period, followed by a decline after the age of 1–2 years. This differs slightly from previous observations, in which the major decline was observed in different age intervals. As for the relative percentages, naive cells had the highest numbers during the newborn period, then showed a gradual decline. By contrast, the memory B cell compartment, the cells with known functions in humoral immunity, autoimmunity, and chronic infectious diseases, demonstrated a gradual increase through life span [8].

In summary, we established the reference values for lymphocyte subsets stratified by age groups. Although this is not a national multicentre study, our study was conducted in one of the reference hospitals of a cosmopolitan city with different ethnic and cultural components. In light of previous literature and statistical analyses, we have reached a relatively large number of healthy participants and achieved our statistical goals with meaningful statistical reliability. We think the data presented can be readily adapted to medical practice.

## Informed consent

Ethical approval was obtained from Marmara University Medical Faculty Clinical Researches Ethics Committee with the number of 09.2015.239/70737436-0.50.06.04. All participants provided informed consent.
